# Understanding pathways from primary health care to universal health coverage outcomes: a realist review

**DOI:** 10.3389/fpubh.2026.1808891

**Published:** 2026-05-28

**Authors:** Neymat Chadha, Devaki Nambiar, Sharmada Sivaram, Dhanashri Bhagal, Sarita Kalundia, Vaibhav Agavane, Urvi Patel, M. S. Navya, Swati Roy, Meike Schleiff, Prashanth N. Srinivas, Sapna Desai, Arnab Mukherji

**Affiliations:** 1 George Institute for Global Health, New Delhi, India; 2 University of New South Wales, Sydney, NSW, Australia; 3 Prasanna School of Public Health, Manipal Academy of Higher Education, Manipal, India; 4 Indian Institute of Management Bangalore, Bengaluru, India; 5 Ekjut, Chakradharpur, Jharkhand, India; 6 Institute of Public Health, Bengaluru, India; 7 University College London, London, United Kingdom; 8 Population Council Institute, New Delhi, India

**Keywords:** community engagement, financial risk protection, health systems strengthening, primary health care, realist review, service coverage, universal health coverage

## Abstract

**Introduction:**

Primary Health Care (PHC) rooted in community empowerment, integrated service delivery, and multisectoral action is central to efforts toward Universal Health Coverage (UHC), yet little is known about how PHC reforms influence UHC outcomes across contexts. This review examines how PHC approaches affect population coverage, service coverage, and financial protection by identifying what works, for whom, under what circumstances, and why.

**Methods:**

A realist review was conducted using studies from countries with the greatest gains in the UHC Service Coverage Index between 2000 and 2021. Forty-two studies met quality criteria following blinded screening, appraisal, and data extraction. Data were synthesized through iterative development of explanatory configurations linking PHC mechanisms, contexts, and UHC outcomes. The evidence base was limited, concentrated mainly in India and China, and largely published after 2020.

**Results:**

Two key configurations emerged: one on reproductive, maternal, newborn, and child health and nutrition, and another on overall PHC design and delivery. The predominance of RMNCH-focused reforms reflects long-standing reform sequencing in many health systems and highlights how RMNCH platforms often function as foundational entry points for PHC strengthening. Across both configurations, reforms frequently emphasized service expansion, benefit package design, and insurance or financing initiatives, with greater attention to contact than effective coverage. Frontline workers, community organizations, and multisectoral actors played critical roles, while persistent geographic, transport, and equity barriers hindered progress. PHC reforms contributed to improvements in coverage and financial protection but fell short in reaching marginalized groups, reducing financial barriers, and enhancing service quality.

**Discussion:**

Strengthening community engagement, regulating private sector roles, and advancing multisectoral collaboration remain essential, and further research is needed to understand how PHC strategies function across diverse contexts to better support UHC.

**Systematic review registration:**

https://www.crd.york.ac.uk/PROSPERO/view/CRD42024523631.

## Introduction

Universal Health Coverage (UHC) is defined as access for all people to a full range of quality services when they need it, where they need it, without financial hardship (1). It owes its legacy to the agenda of Health for All, over which global consensus was achieved back in 1978 with the Alma Ata Declaration on Primary Health Care (PHC) (2). PHC is defined as “whole-of-society approach to health that aims at ensuring the highest possible level of health and well-being and their equitable distribution by focusing on people’s needs and as early as possible along the continuum from health promotion and disease prevention to treatment, rehabilitation and palliative care, and as close as feasible to people’s everyday environment” (3). Strengthening PHC systems and ensuring their efficiency is seen as crucial for advancing Universal Health Coverage and achieving Sustainable Development Goal 3 (SDG3) (4). PHC is the foundation for health systems globally. With its roots going back to the 1978 Alma Ata Declaration as a strategy to achieve universal health by the year 2000, the declaration provided a unified definition and vision for primary health care. More recently, the importance and effectiveness of PHC in maintaining robust health systems was further reaffirmed in the Astana Declaration of 2018 (4–6). Despite the interconnectedness of PHC and UHC, early discussions on UHC from 2005 onwards heavily focused on insurance and financial risk protection. This focus was later questioned and critiqued (7, 8). Critiques highlighted that emphasizing coverage limits UHC to insurance and financial protection, neglecting the necessary focus on health, well-being, and their social determinants (9–11).

Over the years, there has been increasing support advocating PHC reforms as a vital step toward achieving UHC and building a ‘fit-for-purpose’ health system (4, 12, 13). However, there continues to be limited investigation into how strengthening primary health care reforms impact UHC outcomes and how these outcomes differ across various contexts. According to World Health Organization’s operational framework, primary health care comprises of three enmeshed components: integrated health services that incorporate primary care and public health functions, multisectoral policies and actions focusing on addressing broader and upstream determinants of health, and engaging individuals, families and communities to boost social participation and promote self-care and self-reliance in health (2, 3). However, globally, reform efforts have primarily concentrated on integrated service delivery (14–18).

Recent studies underscore that while integrated service delivery has been the dominant focus of primary health care (PHC) reforms, this approach alone is insufficient to achieve Universal Health Coverage (UHC) (15). Instead, PHC must be reconceptualized as a whole-of-society strategy that integrates multisectoral action, community empowerment, and coordinated care across all levels of the health system (3, 15). Despite this broader framing, studies reveal that many countries continue to face governance and structural challenges such as entrenched vertical programs and fragmented service delivery which constrain the implementation of comprehensive PHC reforms (14). To address these constraints, studies highlight the importance of participatory governance and strategic political economy management to ensure meaningful community engagement and alignment of incentives with preventive and promotive health goals (14). In parallel, research on PHC financing shows that reforms must go beyond increasing funding; they must also ensure that resources are equitably distributed, protected at the point of care, and structured to support coordinated, people-centered services (19). A consistent theme across studies is also the central role of Community Health Workers (CHWs), who are shown to be vital for extending care to underserved populations and improving health outcomes (17). However, CHW programs often remain underfunded and poorly supported, facing systemic challenges such as inadequate supplies, limited supervision, and unfair compensation, which undermine their effectiveness and sustainability (17).

While community action or social participation for health establishes participatory governance foundations enabling communities to shape health-system reforms, multisectoral action (MSA) operationalises those reforms by aligning health with non-health sector policies, resources and governance. Lahariya et al. (20) emphasize that interventions targeting adolescent health and well-being and by extension broader PHC reforms require multisectoral partnerships involving education, nutrition, sanitation, social protection and local governance, because many of the determinants of health and access fall outside the clinical domain. They warn that without joint planning, shared resources, aligned policies and decentralized coordination platforms, multisectoral efforts risk fragmentation of services and widening inequities. Complementing this, Singh, Miller and Closser (2024) highlight that at the meso-level in India, successful MSA depends on institutional convening platforms, sustained leadership and stakeholder capacity to engage across sectors (21). Nambiar et al. (22) further show how local self-governance bodies in Kerala act as mobilization hubs that coordinate multi-sectoral actors, integrate health priorities into decentralized planning and negotiate bureaucratic boundaries.

Although the latter two pillars extend beyond the direct scope of the health sector, social participation in health has driven health reforms and multisectoral actions, both of which significantly impact universal health coverage goals like increased population and service coverage and reduced financial hardship (22–24). The global framing of UHC as “leaving no one behind,” has led to detailed consideration of how communities may be involved in and shape the policies and processes of primary health care delivery. The leaving no one behind agenda is further vexed by longstanding concerns about geographies rural, remote, and populations (displaced or moving, facing social and economic barriers) not yet reached by PHC services and the position of these areas and their populations on the path to UHC.

As we approach the last leg of the Sustainable Development Goals era, calls for a focus on PHC reforms at being made across regions (14, 25, 26). Rajan and colleagues point out that political and financial prioritization of PHC remains elusive owing to tensions created between generalist versus specialist care, (poor) services for poor versus health for all, and condition-versus system focused reform (27). The great variation, in phasing, sequencing and areas of focus in primary health care reform (mentioned above), suggests that rather than either-or pathways, there may be a range of pathways in various contexts, that have given rise to approaches in PHC – with varying allocation of resources and undergirding logics or rationales, resulting in various (kinds of) UHC-relevant outcomes.

With a focus on countries that have demonstrated advancement in UHC – here we use standards put out by the WHO as per the UHC-Service Coverage Index (UHC-SCI) scores, we sought to trace the variation and convergence on PHC interventions that appear to assess UHC-relevant outcomes. In order to capture this variation and possible multiplicity of pathways, we employed a realist review.

Realist reviews seek to unpack the relationships between context, mechanism and outcomes (sometimes abbreviated as CMO), i.e., how particular contexts have triggered (or interfered with) mechanisms to generate the observed outcomes” (28). They have been used or adapted to look at the community engagement and multisectoral action pillars of primary health care in particular, as well as other aspects of primary care such as health service brokerage (29–31), The what, how and why of UHC has also been explored using realist approaches (14, 32). A recent review, specifically explored human resource interventions at primary health care level and their link to UHC, finding that familiar actors could help bridge people to services, culturally sensitive or intercultural approaches could be used to develop differentiated strategies of population sub-groups, and ad-hoc, context-based could increase responsiveness and coverage – thus contributing to UHC (33). We wanted to further contextualize review findings and focus instead on individual country pathways and experiences with UHC attainment through PHC.

By drawing out Context(C) - Mechanisms (M) - Outcomes (O) configurations, we aim to: first, specify the characteristics, attributes, and strategies of PHC interventions and their variations across countries; second, analyze studies involving individuals or communities covered by universal health reform packages, state actors, providers, researchers, or other actors associated with healthcare reform in the specified countries, we unpack what primary healthcare-related processes (or mechanisms) have impacted population coverage, service coverage and/or financial risk protection outcomes, and in what contexts.

## Method

### Study design and rationale

This study employed realist review approach to review evidence on Universal Health Coverage (UHC) population, service, and financial risk protection outcomes. The realist approach was chosen as it would allow to understand “what works, for whom, under what circumstances and why” by identifying mechanisms triggered by PHC programs and the relevant contexts to these programs that lead to UHC outcomes.

### Registration and reporting standards

The protocol for this study has been registered with PROSPERO (CRD42024523631). In line with the 2013 RAMESES guidelines (34, 35), a search was undertaken on PubMed, CINAHL, EMBASE, Web of Science, and Global Index Medicus using MeSH and keyword search terms related to combinations of universal health coverage and primary health care terms, time and geographic bounds (Appendix Table 2).

### Development of initial theory of change

**Figure 1:**
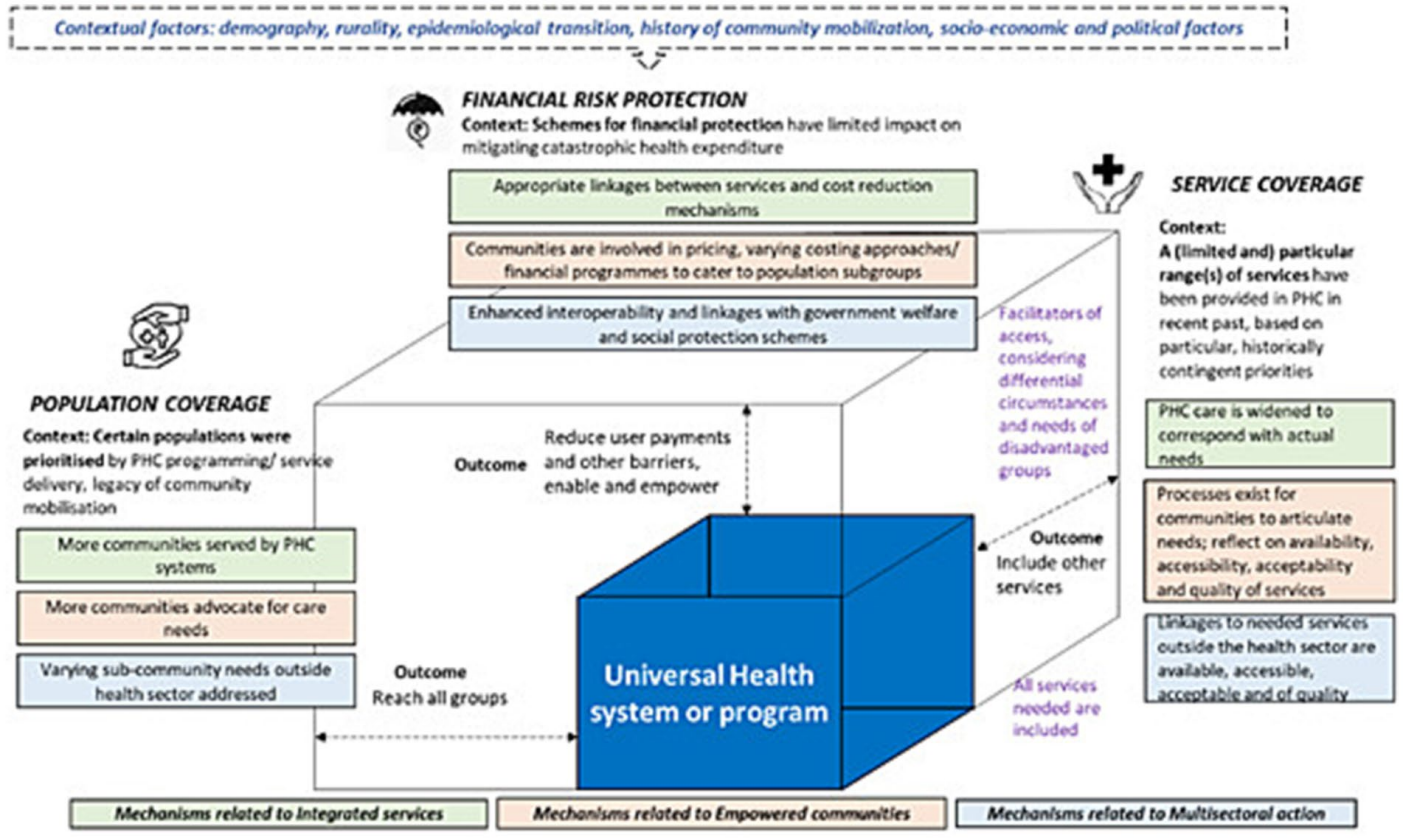
Initial theory of change describing pathways from primary health care to universal health coverage; source: authors.

A series of initial framing workshops were used to develop an initial Theory of Change (ToC) drawing on Tashiro (36), the UHC cube (37, 38), as well as the PHC Monitoring Framework (2), and laid out the possible relationships between PHC and UHC with the scope of developing it further through the realist review, Figure 1).

### Search strategy, scope and setting

The search focused on research or systematic review articles with empirical data (qualitative and/or quantitative) on primary health care services or reform. The countries that were selected had the largest absolute increases in UHC-SCI scores between 2000 and 2021. Countries are treated as comparative cases for theory development rather than focal or exemplary health systems. The study focused on PHC interventions (policies, programs, and community-level interventions) introduced in top decile countries, demonstrating the greatest absolute increases in UHC-SCI scores between 2000 and 2021. These include Thailand, Rwanda, China, Brunei, Darussalam, Cambodia, Senegal, Nepal, Morocco, India, Bangladesh, United Arab Emirates, Bolivia, Vietnam, Cabo Verde, Guyana, Colombia, Seychelles, South Africa, Cyprus and Zambia.

### Rationale for country selection

Although the SCI is widely regarded as a proxy for UHC progress and has demonstrated acceptable sensitivity (39), it has certain limitations. For example, India and Zambia are listed alongside China and Thailand. While India and Zambia have made significant absolute gains, they still face substantial challenges in achieving UHC. In contrast, China and Thailand are reported to have already met the SDG target related to UHC (Sustainable Development Report). Additionally, the absolute index score does not account for inequalities or inequities. As Wagstaff and Neelsen noted, “adjusting the UHC index for inequality in service coverage makes little difference in some countries but reduces it by more than 10% in others” (40). They further indicate that countries like Vietnam, which are included in our list, have focused on financial risk protection and service coverage, yet there is limited understanding of the role of PHC in this index. Day and colleagues highlight the masking of subnational inequalities in national scores (41), while the Global Burden of Disease Sustainable Development Goals collaborators also note that such indices may not provide a clear picture for vulnerable population groups (42). Despite these limitations, we decided to proceed with this proxy for several reasons: India was included in this list along with a justifiable comparison group; other significant UHC and PHC reform achievers in the region, such as Thailand, Nepal, and Bangladesh, were also represented; and important comparators from other regions with different but relevant reform processes, such as China, South Africa, Cambodia, and Rwanda, were reflected in this list.

### Inclusion and exclusion criteria

The inclusion criteria (Appendix Table 1) for the study encompassed research or systematic review articles that presented empirical data, whether qualitative or quantitative, on primary health care services or reforms. Eligible studies had to be published between 2000 and 2024 and focus on countries that showed the largest absolute increases in UHC-SCI scores from 2000 to 2021. Participants in these studies included individuals or communities covered by universal health reform packages, state actors, providers, researchers, or other stakeholders involved in healthcare reform within the specified countries. Conversely, the exclusion criteria ruled out opinion pieces, editorials, or any articles lacking empirical data. While grey literature such as the PRIMASYS case studies provides important contextual insights, only peer-reviewed studies were included to maintain consistency in quality appraisal and methodological transparency (43–46). Studies that did not provide information on population, service, or financial risk protection were also excluded. Additionally, studies that solely offered empirical data on epidemiology at the clinical, facility, or community level in primary health care settings were excluded unless they included other relevant components. Finally, studies focusing exclusively on secondary or tertiary healthcare services were not considered.

### Study selection and screening procedures

Using COVIDENCE software, there was a blinded, double title-abstract and full text screening of items that emerged from the search strategy, with both automated and manual removal of duplicates. Any emergent conflicts from title-abstract and full text screening were resolved by a third independent reviewer. One reviewer extracted bibliometric and descriptive data on study design, geography, authorship, and related variables. Review-specific data were extracted based on preliminary indexing by outcome family, including population coverage, service, and financial risk protection. Additionally, information on contexts and primary health care (PHC) mechanisms was extracted by at least two coders through bilateral meetings and then presented in weekly meetings of the analysis group, which comprised up to 11 members. These meetings aimed to arrive at C-M-O configurations to understand “what works, for whom, under what circumstances and why”. During these meetings, extractions and coding were resolved and reviewed, and decision rules regarding C-M, M-O, and other connections were documented. Coder consensus across at least two-thirds of the membership of each coding meeting was required to confirm judgments. Decisions were recorded in the Excel output generated by COVIDENCE (Tables 1–4).

**Table 1:** Inclusion and exclusion criteria.

Inclusion criteria	Exclusion criteria
Study Type: Research or systematic review articles with empirical data (qualitative and/or quantitative) on primary health care services or reform. Time Frame: Studies published from 2000 to 2024. Geography: We will select countries with the largest absolute increases in UHC-SCI scores between 2000 and 2021. Participants: Studies involving individuals or communities covered by universal health reform packages, state actors, providers, researchers, or other actors associated with healthcare reform in the specified countries.	Opinion pieces, editorials or any articles with no empirical data Studies that did not have information on population, service, or financial risk protection Studies providing empirical data limited to epidemiology (clinical, facility-level or community), in PHCs (if a study has other components – it should be included) Studies that exclusively focused on secondary or tertiary healthcare services.

**Table 2:** Search strategy.

Database	Query	Results
PubMed	Universal Health Insurance”[Mesh]) OR (universal health coverage[Title/Abstract])) OR (universal health care[Title/Abstract])) AND (“Primary Health Care”[Mesh]) OR (primary health care[Title/Abstract])) AND (population coverage[Title/Abstract])) OR (service coverage[Title/Abstract])) OR (financial risk protection[Title/Abstract])) AND ((((((((((((((((((((Thailand) OR (China)) OR (Cambodia)) OR (Nepal)) OR (India)) OR (United Arab Emirates)) OR (Vietnam)) OR (Guyana)) OR (Seychelles)) OR (Cyprus)) OR (Rwanda)) OR (Brunei Darussalam)) OR (Senegal)) OR (Morocco)) OR (Bangladesh)) OR (Bolivia)) OR (Cabo Verde)) OR (Colombia)) OR (South Africa)) OR (Zambia)) AND (2000:2024[pdat])	445
CINAHL	(universal health coverage or universal health care and primary health care) AND TX (population coverage or service coverage or financial risk protection) AND TX (Thailand OR China OR Cambodia OR Nepal OR India OR United Arab Emirates OR Vietnam OR Guyana OR Seychelles OR Cyprus OR Rwanda OR Brunei Darussalam OR Senegal OR Morocco OR Bangladesh OR Bolivia OR Cabo Verde OR Colombia OR South Africa OR Zambia) 2000–2024	129
Embase/ovid	(universal health care or universal health coverage) and primary health care.ab. and population coverage.af.) or service coverage.af. or financial risk protection.af.) and (Thailand or China or Cambodia or Nepal or India or United Arab Emirates or Vietnam or Guyana or Seychelles or Cyprus or Rwanda or Brunei Darussalam or Senegal or Morocco or Bangladesh or Bolivia or Cabo Verde or Colombia or South Africa or Zambia).af. 2000–2024	458
Web of Science	AB = (“universal healthcare”) OR AB = (“universal health coverage”) AND AB = (“primary health care”) AND ALL = (“population coverage”) OR ALL = (“service coverage”) OR ALL = (“financial risk protection”) AND ALL = (Thailand OR China OR Cambodia OR Nepal OR India OR United Arab Emirates OR Vietnam OR Guyana OR Seychelles OR Cyprus OR Rwanda OR Brunei Darussalam OR Senegal OR Morocco OR Bangladesh OR Bolivia OR Cabo Verde OR Colombia OR South Africa OR Zambia) 2000–2024	876
Global Index Medicus	tw:((mh:(“Universal Health Coverage” OR “Universal Health Coverage”) AND (mj:(“Primary Health Care”)) AND la:(“en”)) AND (year_cluster:[2000 TO 2024])) AND (year_cluster:[2000 TO 2024])	4
	tw:((tw:(universal health coverage)) AND (tw:(universal healthcare)) AND (tw:(primary healthcare))) AND (fulltext:(“1” OR “1”) AND la:(“en”)) AND (year_cluster:[2000 TO 2024])	35
	tw:((tw:(universal health coverage)) AND (tw:(universal healthcare)) AND (tw:(primary healthcare)) AND (tw:(financial risk protection))) AND (fulltext:(“1” OR “1”) AND la:(“en”)) AND (year_cluster:[2000 TO 2024])	1
	tw:((tw:(universal health coverage)) AND (tw:(universal healthcare)) AND (tw:(primary healthcare)) OR (tw:(india)) OR (tw:(thailand)) OR (tw:(combodia)) OR (tw:(nepal)) OR (tw:(united aram emirates)) OR (tw:(vietnam)) OR (tw:(morocco)) OR (tw:(bangladesh)) OR (tw:(colombia)) OR (tw:(south africa)) OR (tw:(zambia)) OR (tw:(cyprus)) OR (tw:(rwanda)) OR (tw:(guyana)) OR (tw:(seychelles)) OR (tw:(brunei darussalam)) OR (tw:(bolivia))) AND (fulltext:(“1” OR “1”) AND la:(“en”)) AND (year_cluster:[2000 TO 2024])	136
	tw:((tw:(universal health coverage)) AND (tw:(universal healthcare)) AND (tw:(primary healthcare)) OR (tw:(service coverage)) OR (tw:(financial risk protection)) OR (tw:(population coverage))) AND (fulltext:(“1” OR “1”) AND la:(“en”)) AND (year_cluster:[2000 TO 2024])	136

**Table 3:** Quality appraisal template.

Relevance (Please score between 3 (very good), 2 (criterion met), 1 (criterion partially met), and 0 (criterion is not met) for each criterion)	
Reports country or PHC context	Appraisal category
Provides information on which actors were involved	
Reports PHC process or mechanisms (policies, programs, and community-level interventions)	
Reports at least ONE of our primary outcomes	
Rigor (Please score between 3 (very good), 2 (criterion met), 1 (criterion partially met), and 0 (criterion is not met) for each criterion)	
Objectives AND research questions clearly defined	
Methods, Sampling and Data collection process is explained (you feel you could replicate this study, also say 1 if a reporting standard is used)	
Data analysis explained (and appears sound)	
Results are clearly explained	
There is coherence between all the above	

**Table 4:** Data extraction template.

General information		Extraction category
Study ID	
Title	
Names of all authors	
Corresponding author email	
Single or multi country study	
Country/ies in which study was conducted	Bangladesh, Bolivia, Brunei Darussalam, Cabo Verde (Cape Verde), Cambodia, China, People’s Republic of (also, Hong Kong and Taiwan), Colombia, Cyprus, Guyana, India, Morocco, Nepal, Rwanda, Senegal, Seychelles, South Africa, Thailand, United Arab Emirates, Vietnam, Zambia, Other.
Study funding sources	
Other notes	
Characteristics of included studies	
Health systems classification	WHO PHC category/IES (WHO framework) Service delivery Community engagement Multisectoral Action Other Health Systems Building Block (130 framework) Service delivery (facility based) Service delivery (community-based) Health workforce (facility-based) Health workforce (community based - CHWs, etc.) Medical products, vaccines and technology Financing (including insurance cover of outpatient primary level care) Information learning and accountability Leadership and Governance Community Organizations Societal partnerships
Methods	Aim of study Study design Randomized controlled trial Non-randomized experimental study Cohort study Cross sectional study Case control study Systematic review (including Overview of Reviews) Qualitative research Prevalence study Case series Case report Diagnostic test accuracy study Clinical prediction rule Economic evaluation Text and opinion Mixed Methods Other Years that data was collected Types of knowledge generation primary data collection secondary data analysis evidence synthesis/literature review experiential/tacit knowledge Other Information on analysis (method used) Cut-paste/type information as they describe it Software used Yes No Cannot say Name of software, if used
Participants	Population or beneficiaries description Other actors described Political decision-makers Administrators Community leaders Street level bureaucrats (local leaders/gatekeepers) Community based health providers (eg. frontline providers/outreach staff) Facility based health providers (ie at 1ry level) Referral providers (ie at 2ry 3ry levels, or other services (drug rehab)) Family members and/or caregivers at home Donors Allies/supporters Researchers/Evaluators Other Key actors’ description These are actors involved with implementation, governance, gatekeepers or otherwise mentioned Information provided is for a particular subpopulation or geography particular subpopulation particular geography overall/general population or geography Other Description of particular population or geography, if indicated
Context, mechanisms, outcomes	
Context	Context Details (128, 129) defined as context as (1) preexisting conditions for an intervention, (2) consisting of multiple layers and (3) multiple factors, (4) enabling or disabling of the mechanisms, and (5) through its interaction with mechanisms potentially constituting new contexts (The RAMESES II Project, 2017) Epidemiological context (incidence, prevalence and distribution of health problem/determinants) Social and economic (distribution of social/economic resources among communities of interest) Cultural (beliefs, attitudes and practices of relevant actors including policymakers, practitioners, beneficiaries) Geographic/environmental (features of physical, built or natural environment) Service and organizational (characteristics at meso level related to change, motivation, service environment, training, etc.) Ethical (Extent and nature of equipoise about benefits and harms of approaches) Policy (policy framework, campaigns or initiatives within which work is nested) Political (distribution of power among stakeholders and others involved with design or implementation) Historical (continuing influences of past conditions, sociopolitical relatoinships, policies and frameworks) External shocks and catalytic events (extreme weather, economic crisis, regime change, armed conflict that affect implementation, sustainability, uptake of mechanisms) Does not fit into above Type of context Observable Relational Other
Mechanism	Mechanism Details (RAMESES II) Information on interaction between program resources and the ways that participants interpret and respond (or not) to them Information on relation to context (hidden or otherwise) Explanation of how relates to outcomes Mechanism relationship Iterative with context (i.e., mechanism changes context which then makes more or less of the mechanism happen) Feedback loop with outcome (i.e., outcome also leads to more or less of the mechanism happening) PHC Levers mentioned (WHO-UNICEF Framework) Check all that apply Political commitment and leadership (Strategic) Governance and policy frameworks (Strategic) Funding and allocation of resources (Strategic) Engagement of communities and other stakeholders (Strategic) Models of care (Operational) Primary health care workforce (Operational) Physical infrastructure (Operational) Medicines and other health products (Operational) Engagement with private sector providers (Operational) Purchasing and payment systems (Operational) Digital technologies for health (Operational) Systems for improving quality of care (Operational) Primary health care-oriented research (Operational) Monitoring and evaluation (Operational) Other
Outcomes	Change (specifically increase or greater inclusivity (i.e reduced inequalities)) in populations reached under the ambit of UHC/flagship health scheme Change (specifically increase or greater responsiveness) in the range of services covered (considerations of continuum of care and life course) Change (specifically reduction in OOPE and/or income/employment losses) attributed to health expenditure across all populations (or subpopulations facing disadvantage) Indicators related to Population coverage Enrollment, utilization, coverage of particular age groups or populations considered disadvantaged or “left behind”. Indicators related to Number or types of services covered, expansion of coverage in existing schemes (eg. to cover more aspects, technologies, resourcing, or modes of prevention, treatment, care, palliation, etc.) Indicators related to Change (reduction in OOPE and/or income/employment losses) attributed to health expenditure across all populations (or subpopulations facing disadvantage) Other outcomes

### Quality appraisal

To avoid risk of bias, after full-text screening, the selected studies underwent quality appraisal appropriate to realist approaches (Appendix Table 3). The framework for this was adapted from Hebbar et al. (23). The rigo of the studies was assessed based on a scoring range of 0–5, and the relevance of the studies was analyzed using the same scoring range. A score of 1 was allocated if the criterion was met, and a score of 0 was given if the criterion was not met, meaning if information on a criterion was not adequately provided. Studies with a score of 3 and above for rigor and a score of 3 and above for relevance were included for data extraction. Rigor was assessed based on the following criteria: clearly defined objectives/research questions, explained sampling and overall research approach, detailed data collection process. Throughout screening, there were regular team discussions conducted to try and build shared understanding of eligibility criteria, data analysis, and coherence between objectives, methods, and findings.

**Figure 2:**
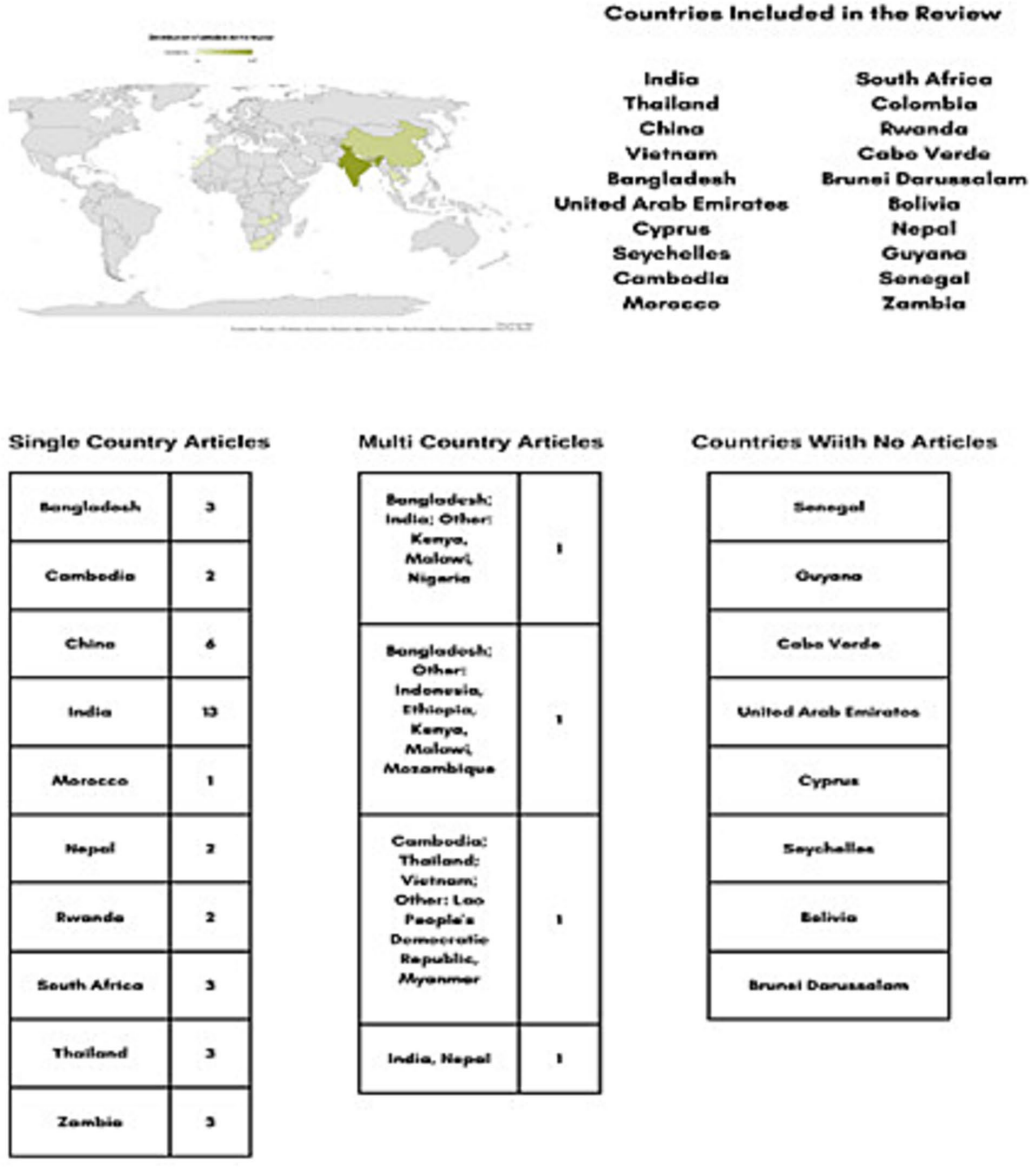
Countries included in realist review of PHC pathways to UHC.

Out of the total 2,192 studies retrieved from different databases, 1,224 studies were screened, and 734 studies were excluded. A total of 488 studies were assessed for eligibility, and 213 studies were excluded. Subsequently, 275 studies were screened for quality appraisal. Only studies that scored at least 12 out of 15 for rigor and 8 out of 12 for relevance were included, resulting in a total of 42 studies being selected (Figure 2).

#### Data synthesis

Data extraction, performed by a single coder, covered descriptives, design, geography, authorship, year, funder, health domain, population, and preliminary indexing of outcomes under population coverage, service coverage, and financial risk protection (Appendix Table 4). The data was then exported from COVIDENCE as an Excel spreadsheet for further coding and analysis.

Four team members (DN, NC, PNS, and VA) through a dialog and discussion derived plausible mechanisms that could explain how a given research publication reports that result. An interim analytical output was produced for the research team examining the underlying individual or collective reasoning as well as related resource availability that could support such a reasoning in line with how mechanisms are understood in realist literature.

### Theory testing and refinement

At a workshop in April 2025, this output was workshopped, re-indexed and re-mapped using an “if, then, because” logic to simplify interpretation and to better integrate wider contexts and inputs into the reform environment. This formulation has been used previously in realist theory-building and synthesis to transparently express program theories as testable causal propositions (47–49). In this configuration, the IF component included programmatic/policy/intervention components of PHC that were introduced by governments, while the THEN included the expected outcomes of such components. The BECAUSE component included mechanisms identified through an interpretive exercise. In realist inquiry, mechanisms are typically reasoning (by the actors/groups of actors in the system) and the related resources introduced into these systems by the intervention which together interact with the context to result in a given outcome (50). Together with other assumptions/factors from the literature that has been included in the component BUT, we derived plausible explanatory pathways/configurations that link PHC investments with UHC outcomes. Authors also arrived at an approach to index and reflect barriers, which were quite prominently described in the literature. This then became the “if, then, because, but” framing used in this paper (51).

## Results

### Characteristics of studies

**Figure 3:**
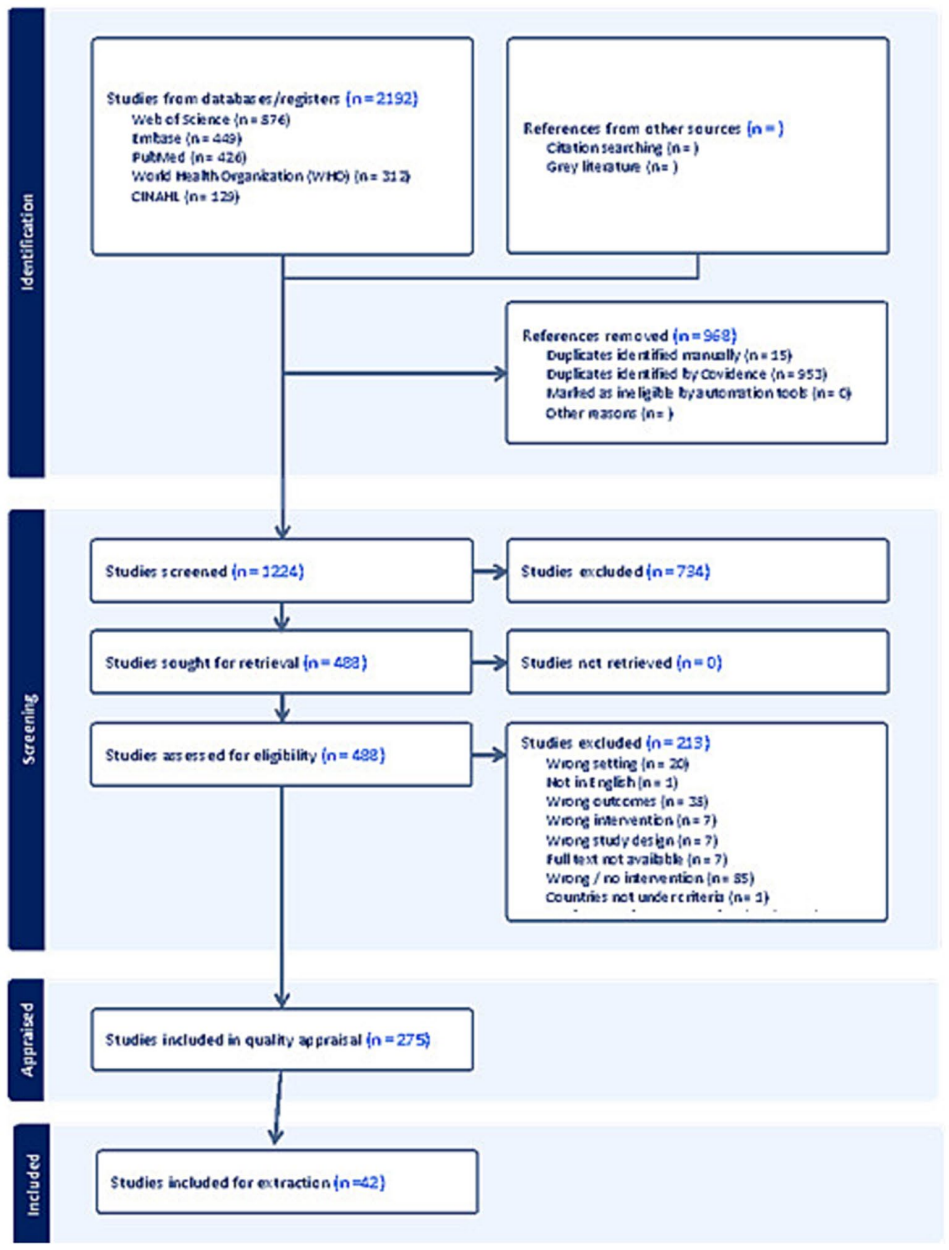
Covidence PRISMA flow chart.

We found a small body of evidence (i.e., only 42 studies) of suitable quality (see Figure 1). Most papers (23 of 42) were published between 2020 and 2024 and were focused on Reproductive, Maternal, Neonatal, Child and Adolescent Health and Nutrition (RMNCAH+N); PHC design and delivery (broader than RMNCAH+N), PHC financing reforms, and PHC human resource reforms. India had the largest representation with 13 studies, followed by China (including Hong Kong and Taiwan) with 6 studies (see Figure 3). Bangladesh, Thailand, South Africa, and Zambia each had 3 studies. Additionally, there were two studies each from Cambodia, Nepal, and Rwanda, and one study each from Morocco and Colombia. Bangladesh was also represented in two additional multi country studies (spanning this and the African region), while India and Cambodia were each included in one additional regional study. Regarding the publication periods, three papers were published between 2005 and 2009, four between 2010 and 2014, 12 between 2015 and 2019, and the majority, 23 studies, were published between 2020 and 2024. Papers from India were published between 2007 and 2023, while those from Thailand were published in 2004, 2015, and 2022.

### Summary of findings

**Figure 4:**
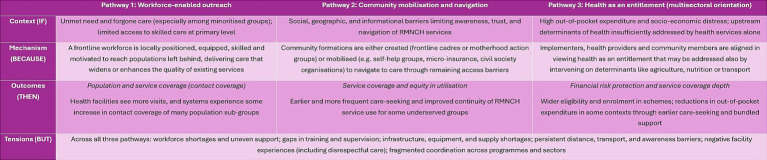
If, then, because, but configuration related to RMNCH and nutrition.

**Figure 5:**
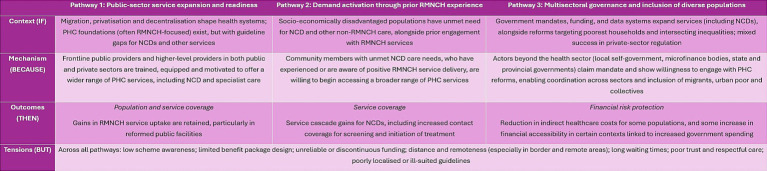
If, then, because, but configuration related to design/delivery of primary health care (PHC).

We arrived at two main configurations, one related to the RMNCH and nutrition domain or subcomponents thereof – seeking to cover women and children as primary populations needing PHC coverage (see Figure 4), and another describing broader PHC reform (in some sense PHC population and service coverage expansion to include more population subgroups and ranges of services, see Figure 5). The following two sections describe these configurations.

We observed that 22 papers (from Bangladesh, China, Colombia, India, Nepal, Morocco, South Africa, Thailand, and Zambia) focused on reproductive, maternal, neonatal, or child health and nutrition and the basics of primary health care (52–73). Nutrition was combined with RMNCH because it was often discussed in the context of RMNCH (For example, the reliance of pregnant women on Anganwadi centers for nutritional support and the role of POSHAN Abhiyan, a flagship national program for improving child and maternal health in India) (59, 62). A large proportion of these studies (*N* = 18) were focused on the service delivery health system building block (52, 53, 56–67, 69, 71–73), while two papers within this group had more of a focus on financing (68, 70), while another two explored human resources (54, 55), although all of the articles had substantial descriptions of the work of a community health workforce or frontline volunteer group.

Figure 4 depicts contextual factors such as unmet healthcare needs for minority groups and high out-of-pocket expenditure on healthcare, causing heightened socio-economic distress (C1a). For instance, gaps in coverage were reported for indicators like skilled attendance at birth in Zambia (71) and India (65), alongside considerable instances of foregone care with iron folic supplementation in India (59) and family planning in Nepal, particularly among ethnic and caste minorities (73). Certain contexts also included the growing prevalence of health burdens such as infertility in Morocco (66). Some of these burdens were not being addressed by health insurance or service scheme design (61, 68, 69). Additionally, high out-of-pocket expenditure and impoverishment risks were observed in multiple settings, including Morocco, Zambia, and India (59, 66, 71) (C1b).

These contextual factors interacted with a series of interventions that had an explicit focus on government programs or schemes for RMNCAH, such as child immunization in India (62), family planning in Nepal (73), postpartum depression in China (56), and overall pregnancy and childcare through the Janani Shishu Suraksha Karyakram (JSSK) in India (61). In most cases, government programs were focused on underserved populations by design – like rural populations, slum populations (C1c). Other studies described specific service model components like kangaroo care (58) or the introduction of new services, such as infertility treatment in Morocco (66). One large study reported on nutrition as a key social determinant and the Poshan Abhiyan as a key pathway for reform in India (59),

As mentioned earlier, almost every study reported substantial reliance on a community health workforce or volunteer group. There was limited reporting on other community engagement mechanisms, although it was noted that “at-home” service outreach and community awareness generation were key mechanisms, for instance in Zambia (72). Patient navigation through ‘health routes’ was reported in Colombia, employing an intercultural approach grounded in planetary boundedness (57). Safe motherhood action groups in South Africa that were gender matched and culturally aligned, were also reported (71).

We identified a range of outcomes associated with these packages (O1a-c). One cluster of outcomes was related to earlier or increased care-seeking, resulting in higher numbers of antenatal or postnatal care visits, immunization coverage and contraceptive use (54, 57, 61, 64, 67, 72). However, this did not always translate to higher population coverage overall (59, 64, 68, 72, 73) or reduced inequities in coverage (52, 64), with the exception of insurance coverage in Thailand (70) and rural community access in Colombia (57). In some cases, we observed reductions in morbidity, for instance, attributable to kangaroo care (58). Additionally, larger packages were linked to reductions in child and maternal mortality in Nepal and India (65) and sustained declines in Colombia (57). Some studies showed declines in out of pocket expenditure owing to early and closer to home care seeking in Bangladesh (53), India (58, 61), and expanded insurance coverage in Rwanda (68) and Thailand (70). Two studies did not report outcomes (56, 63).

Our analysis suggests two micro and one macro level mechanisms that link the aforementioned contexts and outcomes. First, we postulate that the frontline health workforce, locally positioned, adequately equipped, appropriately skilled and thereby well motivated, seeks to reach left behind populations and actively widens the range of services, and the quality of services already available (M1a). Second, it appears that community formations were created (like frontline volunteer cadres or motherhood action groups) or mobilized (like self help groups, micro-insurance schemes or civil society organizations with grassroots linkages) and felt empowered to provide navigation and address access barriers for care-seekers (M1b). Finally, it appears that implementers, health providers and community members have been aligned in viewing health as an entitlement that involves not just healthy system interventions, but also interventions related to nutrition, agriculture and transport (M1c).

Various tensions (or barriers) on the pathway between PHC reform and UHC outcomes were reported in this crop of studies (T1a-e). For one, frontline health workers as well as volunteer groups and community formations faced a range of system and building block shortages in ensuring access to primary care, such as inadequate support, facility human resources, service unavailability, infrastructure gaps, equipment shortages hampered coverage (53, 54, 60, 71). In some cases, gaps in training and professional development for frontline cadres were also reported (59, 67), even as many studies reported a focus on training (13, 63, 65). Negative experiences at health facilities – including experiences of disrespectful behavior were a deterrent to health system users (73), while in others, access barriers like distance, lack of transport and awareness persisted (66).

The second thematic area that emerged in our findings focused on primary health care (PHC) services and system designs (see Figure 5). We found evidence from nine countries: Bangladesh, Cambodia, China, Colombia, India, Rwanda, South Africa, Thailand and Vietnam (71, 74–92). Countries have been pivoting beyond RMNCAH to offer a wider range of services for broader populations. This includes national or subnational flagship programs with strong facility-based service enhancement components (76–81, 83–88, 90, 91), reform of financing and insurance mechanisms (71, 74, 75, 82, 92), as well as urban community health worker programming (89).

The “if” conditions in this configuration were many. The context of migration due to labor, violence, displacement, trafficking, and climate catastrophes is particularly salient in much of East Asia (76, 78) (C2a). Additionally, the region faces issues related to privatization (91) and decentralization (86, 87). The context is also informed by a range of inputs: service enhancements have also been made for existing beneficiary groups, such as non-maternal care for reproductive-aged women (84), mental health (83), urban poor groups (80), care for migrants (76, 78) and the older adult(s) (79), and aligning with legislative reforms related to insurance, such as the 2019 National Health Insurance Bill of South Africa (89) (C2b).

Most reforms and enhancements have focused on the public sector. Additionally, efforts have been made to align governance across different levels of the system within countries, such as local self-government in Kerala, India (86, 87) and across countries in the Greater Mekong Region (76) (C2d). There has been some discussion about attempts to regulate the private sector, with some success in Thailand (91) and weaker regulatory capacity in India (85) (C2e). The use of data for decisionmaking -like use of HMIS and tracking of expenditure - has also been attempted in some contexts (88, 92).

The outcomes issuing from these inputs (the “then” features) were variable. RMNCH gains appeared to be sustained in public facilities in Rwanda, although some groups were still left behind (88) (O2a). In other cases, inequalities were observed based on migratory status, geography, and the level and sector of care. For instance, Kerala’s PHC reforms increased NCD contact coverage, use of outpatient services, especially by women, and engendered trust (86, 87) (O2b). China also experienced improved access to care, although it appears to be concentrated in higher-level facilities (81). In Hong Kong, health literacy gaps persisted, along with long wait times and trust deficits (80). On the financing side, this group of papers suggested that out-of-pocket expenditures (OOPE) remained for groups like migrants, even as they appeared to decline for most in Thailand and China alongside increased government health investment (79, 91) (O2c, O2d).

What mechanisms gave rise to these outcomes? We postulated that there were two micro level factors and one meso level factor connecting contexts and inputs to the outcomes observed in this second group of papers. We postulate that frontline providers in the public sector as well as higher level providers in both public and private sectors had adequate training, equipment, and motivation to offer care for NCDs as well as other forms of specialist care (M2a). Correspondingly, socio-economically disadvantaged community members had unmet NCD care need and had a precedent of positive experiences in the public sector (largely focused on RMNCH+N services), resulting in willingness to access a wider range of services (M2b). Undergirding this was a meso level mechanism of actors beyond the health sector (eg. local self government actors, microfinancing institutions, state governments, private sector regulators, social protection programers) possessing the mandate and willingness to engage with primary health care reforms, offering support for frontline health workers, and including a wide range of beneficiary groups like migrants, self help groups, and the urban poor) (M2c).

As many as seven types of tensions were also flagged in this literature (T2a-g). Low awareness of schemes, their limited package design were associated with low enrolment or utilization in Bangladesh, Cambodia and South Africa (71, 74), while conversely, wider package designs were associated with greater coverage in Rwanda and India (Kerala) (87, 88). Another barrier was seen to be sustained funding, reported in India (87); while in China, funding continuity and increase was seen as an enabler (81). In other cases, barriers were physical distance (82), particularly in border regions (76); long waiting times, lack of trust and respect in treatment course (80), and guidelines ill-suited to purpose in the case of South Africa (90).

## Discussion

Our review yielded two realist-informed configurations for primary health care (RMNCH and nutrition focused, and moving beyond this) across different country contexts. One set appears to reflect what has in many countries been the initial or foregrounded focus of primary health care reforms in many countries – focused on women and children’s health – as these tend to be close to community models of care. The second configuration in many countries posits RMNCH and nutrition services at primary care level as a context and bedrock of further reform, whether focused on service expansion or extension to a wider range population group. It is important to note that in our review, the prominence of evidence from India and China reflects both the scale of PHC reforms and the density of peer reviewed empirical research from these settings, rather than an assumption that the identified mechanisms are unique to large health systems.

We postulated six mechanisms, four at the micro level, one at the meso level and one at the macro level that drive the outcomes seen in the literature. These mechanisms represent context-sensitive explanatory propositions that are observable where particular institutional capacities, political commitments, and frontline delivery arrangements are present. Key actors across these mechanisms are frontline workers and health providers, community members (particularly those facing socio-economic disadvantage), community formations, implementers and facility-level providers, as well as non-health sector actors (17). These actors are the ones negotiating relationships at the community level, at the grassroots, on the frontline. In addition to this, the rationales include positionality (alignment), possession of skill, equipment, motivation and mandate, establishment or co-optation of structures, felt need, as well as trust and willingness to engage (31, 93–96). These are the software elements of system change in primary care that appear to drive reforms forward, toward outcomes that have bearing on UHC. Our literature did mention a lot of barriers, however, including features on the demand side such as physical inaccessibility, trust deficits and negative experiences of careseeking, which are also seen in other contexts such as Saudi Arabia (97). On the supply side, challenges such as limited funding, inadequate training, insufficient compensation, and lack of support for health workers hinder efforts to address workforce shortages and retain staff—especially in underserved areas. These constraints, along with the need to optimize service delivery and overcome upstream barriers like economic conditions and paternalistic practices, continue to impede steady progress on the PHC pathway toward UHC (98, 99).

We can see that in contexts of unmet need and forgone, a focus on left-behind populations, deepening quality and bundling of services while seeking to remove availability and access barriers in the public sector have been a key feature of reforms in many countries. Myriad community strategies like patient navigation, supply and demand side bridging through community health workers, upstream actions to address social determinants have also yielded some gains in terms of utilization – contact coverage and widening of benefit/service packages in primary health care. The dividends in terms of financial risk protection are seen in many cases (though not all). Given that RMNCAH and nutrition have been the traditional province of health systems and a focus of reform particularly in the MDG and SDG periods, the range of mechanisms and their interplay even in individual country contexts are substantial. This domain area represents one where substantial experimentation with PHC mechanisms has taken place and had enough time to effectuate gains at the population health level.

RMNCH-focused PHC reforms appear to function as institutional entry points for broader PHC strengthening by building trust, delivery platforms, and frontline capacity. However, extension to other health challenges requires deliberate redesign of service packages, workforce roles, and financing arrangements rather than passive expansion of existing models. These experiences offer important lessons; however, in many cases, health systems may have initiated or focused their first phase of reforms in this area and therefore there could be cases of “any reform” yielding impacts (100–104), something akin to the “floor effect.” What is less clear is the role of the timing, the duration and the range of experimentation and how this relates to both the types of outcomes seen and tensions reported.

The if/then/because/but configurations for other PHC services mostly build on this first bucket (of RMNCH + Nutrition) services at the primary care level, we also see a substantial emphasis on service delivery components – further expansion of beneficiary groups (to include the older adult(s), urban poor, ward level, migrants etc.) - playing a key role in enhancing inclusion. Widening of service packages into chronic and specialist care also bodes well for inclusion and widening the UHC cube. The scope for these reforms necessarily is wider (going beyond the narrow confines of RMNCAH+), but it is also noteworthy that models of community engagement were not found to be as prolific in this bucket of interventions as in the earlier one. Given the concerns around people-centeredness of care, particularly in disease domains like NCDs (Evaluation of Adoption of People Centered NCD Service Delivery within Primary Health Care in WHO South-East Asia Region, 2014–2021, 2024), this was a noteworthy gap (105).

Given the range of populations covered, multisectoral action has been triggered and, in some cases, regulation of the private sector has been in the purview of reforms (3, 36, 37, 105, 106). Given the predominance of the private sector in many parts of the world, this is a critical element of consideration in the PHC reform process (107) which in turn must be mindful of the great diversity of actors reflected under the banner of “private” (108). This body of evidence suggests that PHC reform – expansion beyond RMNCAH still has a lot of gaps in the face of “new” or multiply minoritized groups being left behind, (over) medicalization of care, growing privatization and the larger continuum of care across a wide(r) range of services. These dynamics warrant closer empirical attention along the path to UHC. The limited empirical visibility of multisectoral action mechanisms likely reflects challenges in attribution, measurement, and sector-specific research silos, rather than an absence of multisectoral engagement in PHC reform processes.

Financing reforms that have been introduced in these contexts in many parts of the world – using insurance mechanisms – are arguably not “primary health” focused enough to even warrant inclusion. However, our synthesis suggests that with wide population coverage and inclusion of outpatient services and bundling of care, particularly following crises or in periods where political commitment to broad based reform may be high, sweeping improvements may be catalyzed (109–112).

This is not consistently or clearly the case, however – we found mixed evidence that risk protection focused interventions can a truly represent reform at primary care level (particularly since they may have a vexed service delivery and community engagement component and therefore not be sufficiently plugged into communities or the health system) and b) deliver even financial risk protection across an entire population. It does appear, however, that a focus on financing reform typically does follow economic or political upheaval – as the opportunity structure for such a kind of change may only emerge then (113–116). What kind of reform and how this relates to PHC remains a bit of a vexed question. At this juncture, moreover, as many countries do indeed have some kind of publicly funded insurance, prepayment or risk pooling mechanism, how this affects service supply and citizen demand may need to be explored further (117).

A major “mechanism” of intervention at the community level is the non-clinical worker–this we found cannot be classified as service delivery alone – but rather has a key element of community engagement in it. Studies we explored found that a number of UHC relevant outcomes–particularly related to availability and acceptability barriers may be addressed, if human resource–community volunteer and worker reforms that lack supportive structures and supply side enhancements can bring populations to bear the brunt of quality and effective coverage gaps (118). We were hoping to derive literature that revealed further insights into how PHC “teams” may function and what this mechanism could yield on the UHC path, but such evidence was not forthcoming in the existing set of studies in this (stage of) review. Experience from other countries, such as Brazil, that have invested in a team care approach, have seen improvements in quality but continue to face challenges with service completeness and the professional practice of those teams (119). This is obviously a key area for further inquiry in PHC focused research.

### Implications for policy

The review suggests that among the single biggest implications of PHC reform have been in the area of workforce. The introduction of community workers – initially with an emphasis on RMNCAH tasks has been quite key in ensuring last mile access. This said, greater consideration of their status and support for their functioning, their integration and rights, is an area that clearly warrants further attention. Another finding is that whether RMNCAH focused reform or otherwise, there has been some success in achieving contact coverage, but both data and programming focused on effective coverage is lacking. Contact coverage refers to service utilization or contact with a health service, whereas effective coverage requires care of sufficient quality, continuity, and appropriateness to produce meaningful improvements in health outcomes. Most studies included in this review operationalized UHC outcomes at the level of contact coverage, which limits inference about effective coverage despite reported gains in access and utilization.

Health systems need to improve their ability to track effective coverage and as much, deliver care to the extent of reducing burden, delaying (multi-)morbidity and mortality, and improving quality of life. Further, as has been the case with immunization with zero-dose (66), identifying population subgroups who face compound vulnerabilities and may be identified for PHC outreach. In some instances, persons being left behind may not be the “usual suspects”- a study in India found, for example that that while women are covered by PHC services (which are largely focused on pregnancy and childbirth), men can end up being excluded from cardiovascular risk screening at the primary care level for a variety of reasons (67). This phenomenon, as well as its relation to existing service delivery design – where community health workers are women – requires further policy attention and, where needed re-programming.

### Implications for research

A clear implication of this survey of the evidence is that the published literature is not likely where much of the story of PHC to UHC pathways resides, is kept or told. Some models and mechanisms are documented but more granular understandings of how various mechanisms fare – including how they are experienced by diverse actors within the same setting remainsa lingering question that our evidence synthesis could not shed adequate light on. In addition, the impact of PHC efforts focused on prevention and promotion, and how they may be operationalized to determine costs burden averted, will be another area of methodological development that we have not seen in the literature in this group of countries.

Importantly, despite widespread implementation of team-based PHC models globally, empirical studies explicitly linking PHC team composition, functioning, and internal coordination to UHC outcomes were scarce in the reviewed literature. This gap appears to reflect limitations in evaluation design and reporting rather than an absence of team-based care in practice, highlighting a priority area for future realist and implementation research focused on PHC team dynamics and their contribution to effective coverage.

## Limitations

We found a circumscribed body of evidence of suitable quality in this review. For one, evidence of quality was not present for a number of geographies that were in our scope. The lack of papers from the United Arab Emirates, Cyprus, Brunei Darussalam, Guyana, Seychelles, Cabo Verde, Bolivia, and Senegal was striking as there have been significant PHC related reforms in a number of these settings (68, 69). Some of these gaps are attributable to a major limitation of our study, which is that our search strategy required reference and framing as “primary health care” (69) where for instance in Senegal, basic health services provided at the community level through a network of health huts and outreach sites are often referred to under the “community health” umbrella (70). A more comprehensive strategy would have allowed for inclusion of such literatures.

We were primarily focused on the peer reviewed empirical literature which excluded a number of important and flagship publications like the PRIMASYS case studies, which exist for a number of our countries of interest (Bangladesh, Colombia, Rwanda, South Africa, Thailand) (120–125). Regional UHC initiatives which include a component of PHC strengthening – largely focused on reproductive maternal and child health in the AMRO region (126) as well as flagship national initiatives on NCD service for UHC, as evidenced in China (78, 81, 101, 127) were also not reflected in the article types and databases sourced for this this review. This restriction was a deliberate methodological choice to ensure consistency in quality appraisal and methodological transparency across a large, multi-country evidence base; as a result, important PHC reform experiences documented primarily in policy reports, program evaluations, and other forms of grey literature, particularly those related to governance arrangements and multisectoral action, may not be fully captured in this synthesis.

Moreover, while PHC related reform may be underway in contexts like Bolivia or UAE, indeed there is other evidence (69) to suggest this is the case -English language academic publications may not be available owing to country prioritization of this as an output, as well as gaps in PHC research infrastructure that may exist in a number of countries (73). References to individual countries may have appeared in reports and editorials, but owing to our exclusion criteria, they are not included in this review. While this is a limitation, it also suggests that more extensive research in these contexts, published in English, could be done, offering not just domestic, but global insights.

Finally, our application of the if-then-because-but framing of realist interpretation requires hypothesizing causal mechanisms from a literature that does not directly report them. This runs the risk of over-interpretation or projection on the part of the coauthors as well as the possibility of alternative interpretations.

## Conclusion

We can see that in contexts of unmet need, mounting burden, emerging challenges of privatization, economic crises (in some cases) and human resource shortages, focusing on specific left-behind populations, deepening quality and bundling of services with a strong community worker backbone, alongside addressal of availability and (financial) access barriers in the public sector have been a key feature of PHC reforms in many countries. These have yielded gains in RMNCAH and overall contact coverage and reductions in impoverishment. Equity gaps do remain, however. A major takeaway is the breadth of roles in primary health care – of expanded frontline worker cadres, of community members, leaders, and well as non-health sector actors, that appear to have played a role in UHC-forward countries. More evidence would yield a more nuanced understanding of the variation of outcomes (across settings but also within a single setting/national context) as well as how mechanisms inter-relate and work together.

## Data Availability

Publicly available datasets were analyzed in this study. This data can be found at: the original datasets can be accessed through the publications cited in the References section. No external data repository or accession numbers apply.
