# Supplementary material for: Understanding pathways from primary health care to universal health coverage outcomes: a realist review
Source: Front Public Health. 2026 May 28;14:1808891. doi: 10.3389/fpubh.2026.1808891 (PMC13254089; doi:10.3389/fpubh.2026.1808891)
Supplement: Supplementary file 1 [file Supplementary_file_1.docx]

**Supplementary File**

**Table 1: Inclusion and exclusion criteria**

| **Inclusion criteria** | **Exclusion criteria** |
| --- | --- |
| Study Type: Research or systematic review articles with empirical data (qualitative and/or quantitative) on primary health care services or reform.  Time Frame: Studies published from 2000 to 2024.  Geography: We will select countries with the largest absolute increases in UHC-SCI scores between 2000 and 2021.  Participants: Studies involving individuals or communities covered by universal health reform packages, state actors, providers, researchers, or other actors associated with healthcare reform in the specified countries. | Opinion pieces, editorials or any articles with no empirical data  Studies that did not have information on population, service, or financial risk protection  Studies providing empirical data limited to epidemiology (clinical, facility-level or community), in PHCs (if a study has other components – it should be included)  Studies that exclusively focused on secondary or tertiary healthcare services. |

**Table 2: Search Strategy**

| **Database** | **Query** | **Results** |
| --- | --- | --- |
| PubMed | Universal Health Insurance"[Mesh]) OR (universal health coverage[Title/Abstract])) OR (universal health care[Title/Abstract])) AND ("Primary Health Care"[Mesh]) OR (primary health care[Title/Abstract])) AND (population coverage[Title/Abstract])) OR (service coverage[Title/Abstract])) OR (financial risk protection[Title/Abstract])) AND ((((((((((((((((((((Thailand) OR (China)) OR (Cambodia)) OR (Nepal)) OR (India)) OR (United Arab Emirates)) OR (Vietnam)) OR (Guyana)) OR (Seychelles)) OR (Cyprus)) OR (Rwanda)) OR (Brunei Darussalam)) OR (Senegal)) OR (Morocco)) OR (Bangladesh)) OR (Bolivia)) OR (Cabo Verde)) OR (Colombia)) OR (South Africa)) OR (Zambia)) AND (2000:2024[pdat]) | 445 |
| CINAHL | ( universal health coverage or universal health care and primary health care ) AND TX ( population coverage or service coverage or financial risk protection ) AND TX ( Thailand OR China OR Cambodia OR Nepal OR India OR United Arab Emirates OR Vietnam OR Guyana OR Seychelles OR Cyprus OR Rwanda OR Brunei Darussalam OR Senegal OR Morocco OR Bangladesh OR Bolivia OR Cabo Verde OR Colombia OR South Africa OR Zambia ) 2000 - 2024 | 129 |
| Embase/ovid | (universal health care or universal health coverage) and primary health care).ab. and population coverage.af.) or service coverage.af. or financial risk protection.af.) and (Thailand or China or Cambodia or Nepal or India or United Arab Emirates or Vietnam or Guyana or Seychelles or Cyprus or Rwanda or Brunei Darussalam or Senegal or Morocco or Bangladesh or Bolivia or Cabo Verde or Colombia or South Africa or Zambia).af. 2000-2024 | 458 |
| Web of Science | AB=("universal healthcare" ) OR AB=("universal health coverage") AND AB=("primary health care" ) AND ALL=("population coverage") OR ALL=("service coverage") OR ALL=("financial risk protection") AND ALL=(Thailand OR China OR Cambodia OR Nepal OR India OR United Arab Emirates OR Vietnam OR Guyana OR Seychelles OR Cyprus OR Rwanda OR Brunei Darussalam OR Senegal OR Morocco OR Bangladesh OR Bolivia OR Cabo Verde OR Colombia OR South Africa OR Zambia) 2000 - 2024 | 876 |
| Global Index Medicus | tw:(( mh:("Universal Health Coverage" OR "Universal Health Coverage") AND ( mj:("Primary Health Care")) AND la:("en")) AND (year_cluster:[2000 TO 2024])) AND (year_cluster:[2000 TO 2024]) | 4 |
|  | tw:((tw:(universal health coverage)) AND (tw:(universal healthcare)) AND (tw:(primary healthcare))) AND ( fulltext:("1" OR "1") AND la:("en")) AND (year_cluster:[2000 TO 2024]) | 35 |
|  | tw:((tw:(universal health coverage)) AND (tw:(universal healthcare)) AND (tw:(primary healthcare)) AND (tw:(financial risk protection))) AND ( fulltext:("1" OR "1") AND la:("en")) AND (year_cluster:[2000 TO 2024]) | 1 |
|  | tw:((tw:(universal health coverage)) AND (tw:(universal healthcare)) AND (tw:(primary healthcare)) OR (tw:(india)) OR (tw:(thailand)) OR (tw:(combodia)) OR (tw:(nepal)) OR (tw:(united aram emirates)) OR (tw:(vietnam)) OR (tw:(morocco)) OR (tw:(bangladesh)) OR (tw:(colombia)) OR (tw:(south africa)) OR (tw:(zambia)) OR (tw:(cyprus)) OR (tw:(rwanda)) OR (tw:(guyana)) OR (tw:( seychelles )) OR (tw:( brunei darussalam)) OR (tw:(bolivia))) AND ( fulltext:("1" OR "1") AND la:("en")) AND (year_cluster:[2000 TO 2024]) | 136 |
|  | tw:((tw:(universal health coverage)) AND (tw:(universal healthcare)) AND (tw:(primary healthcare)) OR (tw:(service coverage)) OR (tw:(financial risk protection)) OR (tw:(population coverage))) AND ( fulltext:("1" OR "1") AND la:("en")) AND (year_cluster:[2000 TO 2024]) | 136 |

**Table 3: Quality Appraisal Template**

| Relevance  (Please score between 3 (very good), 2 (criterion met), 1 (criterion partially met), and 0 (criterion is not met) for each criterion) | |
| --- | --- |
| Reports country or PHC context |  |
| Provides information on which actors were involved |  |
| Reports PHC process or mechanisms (policies, programmes, and community-level interventions) |  |
| Reports at least ONE of our primary outcomes |  |
| Rigour  (Please score between 3 (very good), 2 (criterion met), 1 (criterion partially met), and 0 (criterion is not met) for each criterion) | |
| Objectives AND research questions clearly defined |  |
| Methods, Sampling and Data collection process is explained (you feel you could replicate this study, also say 1 if a reporting standard is used) |  |
| Data analysis explained (and appears sound) |  |
| Results are clearly explained |  |
| There is coherence between all the above |  |
|  |  |

**Table 4: Data Extraction Template**

| **General Information** | |
| --- | --- |
| Study ID |  |
| Title |  |
| Names of all authors |  |
| Corresponding author email |  |
| Single or multi country study |  |
| Country/ies in which study was conducted | Bangladesh, Bolivia, Brunei Darussalam, Cabo Verde (Cape Verde), Cambodia, China, People’s Republic of (also, Hong Kong and Taiwan), Colombia, Cyprus, Guyana, India, Morocco, Nepal, Rwanda, Senegal, Seychelles, South Africa, Thailand, United Arab Emirates, Vietnam, Zambia, Other. |
| Study funding sources |  |
| Other notes |  |
| **Characteristics of included studies** | |
| Health Systems Classification | WHO PHC category/ies (WHO framework)   1. Service Delivery 2. Community Engagement 3. Multisectoral Action 4. Other   Health Systems Building Block (Sacks et al 2019 framework)   1. Service delivery (facility based) 2. Service delivery (community-based) 3. Health workforce (facility-based) 4. Health workforce (community based - CHWs, etc) 5. Medical products, vaccines and technology 6. Financing (including insurance cover of outpatient primary level care) 7. Information learning and accountability 8. Leadership and Governance 9. Community Organisations 10. Societal partnerships |
| Methods | Aim of study  Study design   1. Randomised controlled trial 2. Non-randomised experimental study 3. Cohort study 4. Cross sectional study 5. Case control study 6. Systematic review (including Overview of Reviews) 7. Qualitative research 8. Prevalence study 9. Case series 10. Case report 11. Diagnostic test accuracy study 12. Clinical prediction rule 13. Economic evaluation 14. Text and opinion 15. Mixed Methods 16. Other   Years that data was collected  Types of knowledge generation   1. primary data collection 2. secondary data analysis 3. evidence synthesis/literature review 4. experiential/tacit knowledge 5. Other   Information on analysis (method used)  Cut-paste/type information as they describe it  Software used   1. Yes 2. No 3. Can't say   Name of software, if used |
| Participants | Population or Beneficiaries description  Other actors described   1. Political decision-makers 2. Administrators 3. Community leaders 4. Street level bureaucrats (local leaders/gatekeepers) 5. Community based health providers (eg. frontline providers/outreach staff) 6. Facility based health providers (ie at 1ry level) 7. Referral providers (ie at 2ry 3ry levels, or other services (drug rehab)) 8. Family members and/or caregivers at home 9. Donors 10. Allies/supporters 11. Researchers/Evaluators 12. Other   Key actors’ description  These are actors involved with implementation, governance, gatekeepers or otherwise mentioned  Information provided is for a particular subpopulation or geography   1. particular subpopulation 2. particular geography 3. overall/general population or geography 4. Other   Description of particular population or geography, if indicated |
| **Context, Mechanisms, Outcomes** | |
| Context | Context Details (Booth 2019 and Greenhalgh & Manzano 2022) (defined as context as (1) preexisting conditions for an intervention, (2) consisting of multiple layers and (3) multiple factors, (4) enabling or disabling of the mechanisms, and (5) through its interaction with mechanisms potentially constituting new contexts (The RAMESES II Project, 2017)   \| Epidemiological context (incidence, prevalence and distribution of health problem/determinants) \| \| --- \| \| Social and economic (distribution of social/economic resources among communities of interest) \| \| Cultural (beliefs, attitudes and practices of relevant actors including policymakers, practitioners, beneficiaries) \| \| Geographic/environmental (features of physical, built or natural environment) \| \| Service and organisational (characteristics at meso level related to change, motivation, service environment, training, etc.) \| \| Ethical (Extent and nature of equipoise about benefits and harms of approaches) \| \| Policy (policy framework, campaigns or initiatives within which work is nested) \| \| Political (distribution of power among stakeholders and others involved with design or implementation) \| \| Historical (continuing influences of past conditions, sociopolitical relatoinships, policies and frameworks) \| \| External shocks and catalytic events (extreme weather, economic crisis, regime change, armed conflict that affect implementation, sustainability, uptake of mechanisms) \| \| Doesn't fit into above \|   Type of context   1. Observable 2. Relational 3. Other |
| Mechanism | Mechanism Details (RAMESES II)   \| Information on interaction between program resources and the ways that participants interpret and respond (or not) to them \| \| --- \| \| Information on relation to context (hidden or otherwise) \| \| Explanation of how relates to outcomes \|   Mechanism relationship   1. Iterative with context (i.e. mechanism changes context which then makes more or less of the mechanism happen) 2. Feedback loop with outcome (i.e. outcome also leads to more or less of the mechanism happening)   PHC Levers mentioned (WHO-UNICEF Framework)  Check all that apply   1. 1. Political commitment and leadership (Strategic) 2. 2. Governance and policy frameworks (Strategic) 3. 3. Funding and allocation of resources (Strategic) 4. 4. Engagement of communities and other stakeholders (Strategic) 5. 5. Models of care (Operational) 6. 6. Primary health care workforce (Operational) 7. 7. Physical infrastructure (Operational) 8. 8. Medicines and other health products (Operational) 9. 9. Engagement with private sector providers (Operational) 10. 10. Purchasing and payment systems (Operational) 11. 11. Digital technologies for health (Operational) 12. 12. Systems for improving quality of care (Operational) 13. 13. Primary health care-oriented research (Operational) 14. 14. Monitoring and evaluation (Operational) 15. Other |
| Outcomes | \| Change (specifically increase or greater inclusivity (i.e reduced inequalities)) in populations reached under the ambit of UHC/flagship health scheme \| \| --- \| \| Change (specifically increase or greater responsiveness) in the range of services covered (considerations of continuum of care and life course) \| \| Change (specifically reduction in OOPE and/or income/employment losses) attributed to health expenditure across all populations (or subpopulations facing disadvantage) \| \| Indicators related to Population coverage Enrollment, utilisation, coverage of particular age groups or populations considered disadvantaged or “left behind.” \| \| Indicators related to Number or types of services covered, expansion of coverage in existing schemes (eg. to cover more aspects, technologies, resourcing, or modes of prevention, treatment, care, palliation, etc.) \| \| Indicators related to Change (reduction in OOPE and/or income/employment losses) attributed to health expenditure across all populations (or subpopulations facing disadvantage) \| \| Other outcomes \| |
